# Determinants of low birth weight among newborns delivered at Tirunesh Beijing General Hospital, Addis Ababa, Ethiopia: a case-control study

**DOI:** 10.1186/s12884-021-04275-6

**Published:** 2021-11-27

**Authors:** Mesfin Tadese, Abdulwahhab Seid Minhaji, Chalachew Tegegne Mengist, Fetene Kasahun, Getaneh Baye Mulu

**Affiliations:** 1 Department of Midwifery, College of Health Sciences, Debre Berhan University, Debre Berhan, Ethiopia; 2 Department of Nursing, College of Health Sciences, Debre Berhan University, Debre Berhan, Ethiopia

**Keywords:** Low birth weight, Determinants, Case-control, Ethiopia

## Abstract

**Background:**

Low birth weight is weight less than 2500 g or 5.5 lb. at birth. Globally, more than 20 million infants (15-20%) are born with a low birth weight each year. Birth weight is the primary indicator of the health status of neonates and is the primary factor that determines the infant’s physical, survival, and mental growth. Thus, the study aimed to investigate the determinants of low birth weight among newborn babies delivered at Tirunesh Beijing General Hospital, Addis Ababa, Ethiopia.

**Methods:**

We performed a facility-based unmatched case-control study among 453 (151 cases and 302 controls) deliveries conducted at Tirunesh Beijing General Hospital. Birth records and maternal antenatal care (ANC) files were reviewed from March 1 to April 30, 2019. Consecutive sampling was employed to select study participants. Data were entered into Epi-data version 4.2.1 and analyzed using SPSS version 25 statistical software. Descriptive statistics and logistic regression analysis were computed to identify independent determinants of low birth weight. A *p*-value of ≤0.05 was used to declare statistical significance.

**Result:**

Four hundred fifty-three birth records of babies (151 cases and 302 controls) were reviewed**.** Women who reside in rural area [AOR (CI) = 3.12 (1.63-5.98)], being merchant [(AOR (CI) = 2.90 (1.03-8.22)], danger sign during pregnancy [(AOR (CI) = 4.14 (1.68-10.2)], and maternal weight during pregnancy [(AOR (CI) = 4.94 (3.26-7.52)] were found to be a significant determinants of low birth weight.

**Conclusion:**

Residence, occupation, danger signs, and maternal weight during pregnancy were significant determinants of low birth weight. Socioeconomic development, early detection and management of complications, and encouraging nutrition and weight during pregnancy are crucial for minimizing the risk of delivering low birth weight babies.

## Background

The birth weight of a newborn baby is the first weight record, preferably taken within an hour of birth. Birth weight is a good summary of multifaceted public health problems, including long-term maternal malnutrition, chronic illness, and poor health care during pregnancy [1]. World Health Organization (WHO) defines low birth weight (LBW) as the weight of a newborn baby below 2500 g at birth regardless of the gestational age. Further classification of low birth weight includes; low birth weight (below 2500 g), very low birth weight (below 1500 g), and extremely low birth weight (below 1000 g) [2].

Every year more than 20 million babies are born weighing below 2500 g. Globally, this figure accounts for 17% of total birth in low-income countries, a rate more than twofold that of developed countries (7%) [2]. For example, the prevalence of low birth weight in Ghana, Burkina Faso, Senegal, Malawi, and Uganda was 10.2, 13.4, 15.7, 12.1, and 10%, respectively [3]. In Sub-Saharan Africa, the burden of low birth weight was 9.6% [4]. A cross-sectional study in Ethiopia revealed a 12.5% prevalence of low birth weight in Butajira [5] and 15.8% in Wolaita Sodo [6].

Low birth weight babies are highly vulnerable to death during their early childhood periods. Even those who escaped death in their early months and years are still prone to a high incidence of chronic diseases like type II diabetes mellitus, cardiovascular disease, hypertension, immunodeficiency, and impaired language development in their adulthood and old age. It also hurts their reproductive outcomes, i.e., preterm and low birth weight, and neurocognitive development such as intellectual disability, learning impairment, cerebral palsy, retinopathy, and mental retardation [7].

A baby’s birth weight is substantially influenced by the mother’s early life, including fetal growth, childhood and adolescent nutrition, hereditary, and body composition during conception [7]. Other predictors of LBW include socioeconomic status, parity, mid-upper-arm-circumference, insufficient gestational weight gain, lack of antenatal care (ANC) follow-up, preterm labor, medical and obstetric complications, physical activity, smoking, and bad obstetric history [3, 8, 9].

According to the Ethiopian Demographic Health Survey, the burden of low birth weight in Ethiopia was not decreased significantly, 11% in 2011 and 13% in 2016 [1, 10]. This might be attributed to insufficient improvements in the quality of health care services, including delivery at health facilities, ANC coverage, postnatal services, and vaccination [11]. Maintaining good nutrition and healthy weight gain, especially at the beginning of conception, controlling pre-existing medical and obstetric illnesses, increasing intake of folic acid found in fruits, whole grains, and vegetables, good pre-conceptional care and ANC follow-ups, and improving the living standards of the general population were the tips to lower the risks of low birth weight [12].

In Ethiopia, LBW is still a significant public health concern. The attainment of a sustainable development goal is also intensely affected by the progress in newborn deaths. The government of Ethiopia has appreciated the depth of the problem, and nowadays, some changes are being employed by governmental, non-governmental organizations (Columbia University International Center for AIDS Care and Treatment Program (CU-ICAP), WHO), and professional associations like Ethiopian pediatrics society. Evidence-based intervention is required to reduce the burden of the problem and improve newborns’ survival. Observational data on the incidence and determinants of low birth weight are essential for designing interventional activities, particularly in Sub-Saharan Africa [13]. Thus, the study aimed to assess the determinants of low birth weight among newborn babies delivered at Tirunesh Beijing General Hospital, Addis Ababa, Ethiopia.

## Methods

### Study area, period, and design

A facility-based unmatched case-control study was conducted in Tirunesh Beijing General Hospital, Addis Ababa, from March 1 to April 30, 2019. The hospital is found South of Addis Ababa, the capital city of Ethiopia. The town contains ten sub-cities and 116 woredas. It has 12 governmental and nine non-governmental hospitals. Tirunesh Beijing General Hospital is one of the famous public general hospitals providing a range of medical, maternal, and child health services, including delivery services and various inpatient and outpatient healthcare services for more than four hundred thousand catchment populations surrounding the Oromia region.

### Population

In the preceding year, all mothers delivered in Tirunesh Beijing General Hospital were the source population. There were 3798 deliveries in the past year. Cases were those newborns with low birth weight (LBW) (weight of < 2500 g) and controls (Normal Birth Weight) were those live births weighing ≥2500 g and those delivered on the same day as enrolled regardless of the mode of delivery. All singleton births delivered after 28 weeks of gestation or weight of at least 1000 g, and with available information on birth weight in the medical records were included. Newborns with twin or multiple births, visible congenital deformity, i.e., hydrocephalus, stillbirths, birth weight ≥ 4000 g, and incomplete data were excluded from the study.

### Sample size determination

The sample size was determined using the Open Epi version 3.03 statistical software package. The following considerations were made: percent of controls exposed, odds ratio, confidence level 95%, power 80%, and the ratio of controls to cases 2 (Table 1). By adding 5% for incomplete or missed data, the largest sample size becomes 482 (161 cases and 321 controls).

**Table 1:** Sample size calculation for determinants of low-birth-weight among women delivered at Tirunesh Beijing General Hospital, Addis Ababa, Ethiopia

Variables	Percent of controls exposed	Adjusted odds ratio	Sample size Cases/controls [Total]	References
Gestational age	55.8	5.32	31/63 [94]	[14]
Chronic diabetes	85.7	0.27	29/59 [88]	
Signs of pregnancy complication	48.3	2.7	61/122 [183]	
Sex of newborn	13.5	2.1	**161/321 [482]**	[15]
Maternal age	51.2	3.1	50/101 [151]	[16]
Residence	51.9	2.1	106/211 [317]	
Maternal education	3.9	6.0	57/113 [170]	
Folate supplementation	29	5.48	22/44 [66]	[17]

### Variables of the study

Birth weight was the outcome variable. Socio-demographic factors (i.e., maternal age, educational level, occupation, marital status, and residence), maternal and obstetrics characteristics (i.e., weight during pregnancy, gravidity, parity, history of abortion, gestational age at birth, and ANC follow up), and complications during pregnancy, (i.e., chronic hypertension, pregnancy-induced hypertension, and danger sign of pregnancy) were the exposure variables.

### Measurements

#### Birth weight

The weight of the newborns measured within the first 15 min of birth [15].

#### Normal birth weight

Weight at birth between 2500 g and 4000 g.

#### Danger signs of pregnancy

Any one or more early or late pregnancy bleeding, leakage of amniotic fluid, reduced fetal movement, severe headache, convulsion, blurred vision, fever, and severe abdominal pain [14].

#### Gravidity

The number of times a woman has been pregnant, whether a term, live births, stillbirth, abortion, ectopic, or molar pregnancy.

#### Parity

The number of times a woman gives birth to a fetus with a gestational age of 28 weeks or more, regardless of the outcome.

#### Iron and folic acid supplementation

For this study, mothers who supplemented and took iron and folic acid for at least 3 months were considered as “Yes.”

#### Merchant

Women trades in commodities produced by other people, i.e., vegetables (onion, tomatoes, cabbage, potato), plastics (bucket, seat), clothing (trousers, underwear, shoes, t-shirt,) metals (metallic pot, teapot).

#### Maternal weight

The data were derived from four weight measurements in pregnancy from antenatal care registration books, and the average weight was considered for analysis.

### Data collection tools and procedure

A structured and pre-tested checklist was used to extract the maternal antenatal care (ANC) files and birth records. The checklist was designed from the Demographic Health Survey questionnaire and other similar literature works [9, 14, 18]. The checklist consists of socio-demographic, maternal, and neonatal factors. Socio-demographic characteristics, maternal and obstetrical variables were extracted from birth records, while pregnancy complications were obtained from the ANC records. Newborn features, including birth weight and gender, were extracted from the birth records. At birth, the gestational age was calculated using the last normal menstrual period (LNMP) and a review of the maternal history for early ultrasound examination and urine test.

Four trained midwives and one supervisor participated in the data extraction. Consecutive sampling was employed to select cases and controls. For each case (low birth weight) record, two consecutive controls (normal birth weight) records were set and reviewed starting from the data collection date.

### Data quality control

The questionnaire was first prepared in English and later translated to the local language, Amharic, and then back to English by an independent translator to keep the data collection instrument consistent. The tool was first pre-tested on 5% of the samples (8 cases and 15 controls) in Zewditu Memorial Hospital before the actual date of data collection. A necessary adjustment was considered following the result of the pre-test. A one-day training has been given concerning the objective, instrument, and data collection procedure for both data collectors and supervisors. The supervisor and the principal investigators checked and reviewed the collected data for clarity, completeness, and accuracy.

### Data management and analysis

The data were coded, cleaned, and edited. Errors identified during the data cleaning were adjusted after reviewing the original data using the code numbers. Data were entered into Epi-Data version 4.2.1 and analyzed using SPSS version 25 statistical software. The descriptive statistics were summarized and presented using frequency tables, means, standard deviation, and percentages. When the frequencies became smaller, re-categorization/recoding of variables was done. Variables with a *p*-value of ≤0.25 in the bivariable logistic regression were selected for the final model. Multivariable logistic regression models were run to identify independent determinants of LBW. The level of multi-collinearity was checked and fitted using variance inflation factor and tolerance. An adjusted odds ratio (AOR) with a 95% confidence interval (CI) was reported to describe the strength of association. The Omnibus test and Hosmer-Lemeshow goodness-of-fit were applied to check for model fitness. A statistically significant level was stated at a *p*-value of < 0.05.

## Result

### Baseline characteristics

A total of 453 (151 cases and 302 controls) birth records and antenatal care files were reviewed. The mean (±SD) age of cases and controls were 29.17 ± 7.12 and 27.22 ± 5.59, respectively. Twenty-nine (6%) records were incomplete and excluded from the analysis. About 58.3% of cases and 77.5% of controls were within 20 – 35 years. Eighty-six (57%) cases reside in rural areas, while two hundred ten (69.5%) of controls were urban residents. Around 33.1% of cases and 27.5% of controls had attended primary school, and 40.6% of the case group and 21.2% of the control group mothers were within the weight range of 50 – 60 kg during their recent pregnancy. Further, nearly half of mothers in cases (49.7%) and controls (50.7%) were housewives (Table 2).

**Table 2:** Baseline characteristics of women delivered at Tirunesh Beijing General Hospital, Addis Ababa, Ethiopia

Variables	Category	Birth Weight, n(%)		***P***-value
		Cases (151)	Controls (302)	
**Age of the mother**	< 20 years	17 (11.2)	31 (10.3)	0.000®
	20–34 years	88 (58.3)	234 (77.5)	
	≥35 years	46 (30.5)	37 (12.2)	
**Residence**	Urban	65 (43.0)	210 (69.5)	0.000®
	Rural	86 (57.0)	92 (30.5)	
**Religion**	Muslim	27 (17.9)	65 (21.5)	0.702*
	Orthodox	90 (59.6)	166 (55.0)	
	Protestant	31 (20.5)	62 (20.5)	
	Others^a^	3 (2.0)	9 (3.0)	
**Educational status**	No formal education	49 (32.5)	68 (22.5)	0.016®
	Primary	50 (33.1)	83 (27.5)	
	Secondary	28 (18.5)	80 (26.5)	
	Higher education	24 (15.9)	71 (23.5)	
**Occupation**	Employed	26 (17.2)	93 (30.8)	0.001®
	Merchant	38 (25.2)	42 (13.9)	
	Housewife	75(49.7)	153 (50.7)	
	Others^b^	12 (7.9)	14 (4.6)	
**Ethnicity**	Amhara	61 (40.4)	113 (37.4)	0.168*
	Oromo	80 (53.0)	157 (52.0)	
	Tigre	9 (6.0)	18 (6.0)	
	Others^c^	1 (0.7)	14 (4.6)	
**Maternal weight during pregnancy**	50–60 kg	61 (40.6)	64 (21.2)	0.000®
	61–70 kg	58 (38.7)	105 (34.8)	
	71–80 kg	25 (16.7)	101 (33.4)	
	≥81 kg	6 (4.0)	32 (10.6)	

^a^Catholic, Seventh-day Adventist

^b^Daily laborer, waiter, tailor

^c^Silte, Gurage, Afar

®Pearson chi-square

*Fisher’s exact test

### Obstetrics, antenatal, and newborn characteristics

The mean (±SD) birth weight was 2133.5 ± 332.88 g for the low-birth-weight newborns and 3080 ± 360.93 g for the normal birth weight newborns. About 66.3% of cases and 55% of controls were multiparas, and 83.4% of cases and 89.4% of controls were supplemented with iron during pregnancy. The majority of the case group (86.1%) and control group mothers (91.4%) had antenatal care (ANC) follow-up. Female newborns accounted for the more significant proportion of cases (55.0%) and controls (51.7%). Regarding complications during pregnancy, 5.3, 19.9, and 31.8% of case group mothers had chronic hypertension, pregnancy-induced hypertension, and danger signs of pregnancy, respectively. Additionally, five (3.3%) of mothers with cases had a history of low birth weight, and twelve (7.9%) of mothers had a history of abortion (Table 3).

**Table 3:** Obstetrics, antenatal, and newborn characteristics of mothers delivered at Tirunesh Beijing General Hospital, Addis Ababa, Ethiopia

Variables	Category	Birth Weight, n (%)		***P***-value
		Cases (151)	Controls (302)	
**Gravidity**	Primigravida	64 (42.4)	115 (38.1)	0.377®
	Multigravida	87 (57.6)	187 (61.9)	
**Parity**	Primipara	21 (20.2)	72 (38.1)	0.203®
	Multipara	69 (66.3)	104 (55.0)	
	Grand multipara	14 (13.5)	13 (6.9)	
**ANC visit**	Yes	130 (86.1)	276 (91.4)	0.081®
	No	21 (13.9)	26 (8.6)	
**Iron and folic supplementation**	Yes	126 (83.4)	269 (89.4)	0.073®
	No	25 (16.6)	32 (10.6)	
**Hemoglobin**	< 11 g/dl	6 (4.0)	5 (1.7)	0.192*
	≥11 g/dl	145 (96.0)	297 (98.3)	
**Chronic hypertension**	Yes	8 (5.3)	3 (1.0)	0.008*
	No	143 (94.7)	299 (99.0)	
**Pregnancy-induced hypertension**	Yes	30 (19.9)	11 (3.6)	0.000®
	No	121 (80.1)	291 (96.4)	
**Danger signs during pregnancy**	Yes	48 (31.8)	34 (11.3)	0.000®
	No	103 (68.2)	268 (88.7)	
**History of LBW**	Yes	5 (3.3)	3 (1.0)	0.124*
	No	146 (96.7)	299 (99.0)	
**History of abortion**	Yes	12 (7.9)	25 (8.3)	0.903®
	No	139 (92.1)	277 (91.7)	
**Gestational age**	< 37 weeks	105 (69.5)	10 (3.3)	0.000*
	37–42 weeks	43 (28.5)	284 (94.0)	
	> 42 weeks	3 (2.0)	8 (2.7)	
**Sex of the newborn**	Male	68 (45.0)	146 (48.3)	0.550®
	Female	83 (55.0)	156 (51.7)	
**Birth defect**	Yes	6 (4.0)	4 (1.3)	0.091*
	No	145 (96.0)	298 (98.7)	

®Pearson chi-square

*Fisher’s exact test

### Determinants of low birth weight

Bivariable logistic regression analysis was computed, and variables with a *p*-value of ≤0.25 were selected for the multivariable logistic regression analysis model. In the multivariable logistic regression analysis, residence, occupation, danger signs, and maternal weight during pregnancy have shown a statistically significant association with low birth weight (Table 4).

**Table 4:** Determinants of LBW in Tirunesh Beijing General Hospital, Addis Ababa, Ethiopia

Variables	Category	COR (95% CI)	AOR (95% CI)	***P***-value
**Age of the mother**	< 20 years	1	1	
	20–35 years	0.69 (0.36–1.30)	0.41 (0.07–2.53)	0.337
	≥36 years	2.27 (1.09–4.72)	2.45 (0.38–15.8)	0.348
**Residence**	Urban	1	1	
	Rural	3.02 (2.02–4.53)	**3.12 (1.63**–**5.98)**	**0.001**
**Educational status**	No formal education	2.13 (1.18–3.85)	0.88 (0.32–2.43)	0.802
	Primary	1.78 (0.99–3.19)	0.59 (0.21–1.69)	0.330
	Secondary	1.04 (0.55–1.95)	0.68 (0.25–1.85)	0.448
	Higher education	1	1	
**Occupation**	Employed	1	1	
	Merchant	3.24 (1.75–6.00)	**2.90 (1.03**–**8.22)**	**0.045**
	House wife	1.75 (1.05–2.94)	1.13 (0.46–2.77)	0.794
	Others^a^	3.06 (1.26–7.43)	1.58 (0.35–7.07)	0.551
**Parity**	Primipara	1	1	
	Multipara	3.27 (1.28–4.04)	2.28 (0.99–4.75)	0.237
	Grand multipara	3.69 (1.50–9.06)	2.20 (0.68–7.15)	0.189
**ANC visit**	Yes	1	1	
	No	1.72 (0.93–3.16)	0.41 (0.12–1.45)	0.166
**Iron supplementation**	Yes	1	1	
	No	1.67 (0.95–2.93)	1.13 (0.34–3.81)	0.839
**Hemoglobin**	< 11 g/dl	2.46 (0.74–8.19)	1.11 (0.16–7.46)	0.918
	≥11 g/dl	1	1	
**Chronic hypertension**	Yes	5.58 (1.46–21.3)	1.46 (0.18–11.7)	0.724
	No	1	1	
**Pregnancy-induced hypertension**	Yes	6.56 (3.18–13.5)	1.88 (0.60–5.86)	0.277
	No	1	1	
**Danger signs during pregnancy**	Yes	3.67 (2.24–6.02)	**4.14 (1.68**–**10.2)**	**0.002**
	No	1	1	
**History of LBW**	Yes	3.41 (0.81–14.5)	3.01 (0.16–57.8)	0.465
	No	1	1	
**Maternal weight during pregnancy**	50–60 kg	5.08 (1.97–13.0)	**4.94 (3.26**–**7.52)**	**0.000**
	61–70 kg	2.95 (1.16–7.46)	**6.42 (2.46**–**9.13)**	**0.001**
	71–80 kg	1.32 (0.49–3.50)	2.57 (0.65–10.3)	0.181
	≥81 kg	1	1	
**Birth defect**	Yes	3.08 (0.86–11.1)	3.18 (0.38–26.4)	0.283
	No	1	1	

Chi-square test was performed

*LBW* Low Birth Weight

^a^Daily laborer, waiter, tailor

The odds of low birth weight among rural residence were three times higher than the urban counterparts (AOR (CI) = 3.12 (1.63-5.98). The risk of low birth weight was also significantly higher among merchant mothers compared to those employed (AOR (CI) = 2.90 (1.03-8.22). Furthermore, mothers who had a danger sign during pregnancy were four times more likely to deliver low birth weight babies compared to those who have no danger signs (AOR (CI) = 4.14 (1.68-10.2). Mothers whose pregnancy weight ranges from 50 to 60 kg were approximately five times more likely to have low birth weight neonates, compared to those whose weight exceeds 81 kg (AOR (CI) = 4.94 (3.26-7.52).

## Discussion

Low birth weight continues to be a significant cause of neonatal morbidity and mortality. In this study, residence, occupation, danger signs, and maternal weight during pregnancy were significant associates of low birth weight.

The odds of low birth weight were higher among mothers from rural residents than their urban counterparts. This finding was consistent with past studies conducted in India [19], Malaysia [8], Dilla town [17], and Bale Zone Hospitals [15]. This could be due to the difference in education, income, and workload. Women from rural areas had significantly lower levels of education. The higher level of education among urban women provides higher household incomes through securing occupations in a professional field and administrative roles. The rural women were actively involved in domestic and outside activities, and they bear double, particularly in Sub-Saharan Africa, including Ethiopia [8]. These low socioeconomic statuses of rural women restrict access to information and health services, antenatal care follow-ups, danger signs of pregnancy, nutritional status, and fetal growth, thus increasing the risk of low birth weight [20].

However, this finding was in contrast with the report from Jimma Zone, Southwest Ethiopia, where the risk of low birth weight was significantly higher among urban residents [21]. This may account for the variations in study design, data collection technique, and study population. For example, in this study, rural women accounted for more significant cases (57%). But in the latter survey, urban women accounted for more cases (32.6%) than rural (17.1%). In addition, urban dwellers were more likely to experience high-risk lifestyles and behaviors, i.e., smoking, khat chewing, and alcohol drinking, that might have resulted in low birth weight.

This study revealed that the risk of low birth weight was significantly higher among merchant mothers. Our finding is concordant with Snijder et al.’s finding, which documented a significant association between being a merchant and low birth weight [22]. This could result from work-related stress and involvement in strenuous activities or heavy physical work. Additionally, they spend most of their time in business or wholesale trade and may not have enough time to care for themselves. Further, such activities might require prolonged standing. In turn, this increases the action of the sympathetic nervous system in the active muscles and results in backflow of blood from visceral arteries to the active muscles, which results in increased sweating, reduced plasma volume, decreased blood perfusion to uterine and placental arteries, thereby reducing the oxygen and nutrients supply to the fetus [22].

Danger signs during pregnancy were also significantly increased the odds of low birth weight. According to this study, mothers who had a danger sign during pregnancy were four times more likely to deliver low birth weight babies. This finding was supported by previous studies done in India [19], Debre Berhan [14], Amhara regional state referral hospitals [23], and Bale zone [15]. The danger signs and symptoms of pregnancy indicate some kind of complication that adversely affects the growth of the fetus. For instance, hypertensive disorders of pregnancy may cause pre-eclampsia, resulting in reduced perfusion and nutrient and oxygen supply to the fetus, resulting in low birth weight or fetal death [23]. Thus, it is recommended that pregnant women be aware of this sign and timely report/seek medical care. In addition, regular antenatal care follow-up helps early detection and management of these disorders during pregnancy.

Moreover, maternal weight during pregnancy was found to be another determinant factor for low birth weight. Mothers whose pregnancy weight ranges from 50 to 60 kg were more likely to deliver low birth weight babies than those whose weight exceeds 81 kg. This finding was consistent with a previous study in the context of five African countries: maternal underweight significantly increased the risk of low birth weight [3]. A similar result was also represented in China [9], India [19], Bale zone [15], and Jimma [21]. Anthropometric measurements are indicators of maternal nutritional status during pregnancy. In this case, maternal underweight shows chronic malnutrition, which impairs the growth and development of the fetus. Inadequate dietary intake during pregnancy has been shown to reduce the placental weight and surface area, limiting oxygen and nutrient transfer from the placenta to the fetus. Furthermore, poor maternal nutrition may decrease the serum concentrations of hormones, i.e., leptin and estrogen, which results in fetal growth impairment [11]. Therefore, it is recommended for pregnant women to have an adequate diet during pregnancy, particularly for its inter-generational effects [3].

Over 69% of women in the case group delivered before 37 completed weeks of gestation compared to only 3% in the control group. Similarly, in Brazil, prematurity increased the odds of LBW ten times [24]. In addition, a study in Amhara region referral hospitals found a significantly low birth weight among preterm deliveries [23]. This could be because many of the baby’s organs and basic body systems are not fully matured. The earlier the baby was born, the higher the risk of low birth weight.

### Limitation of the study

Since secondary data were used, some potential risk factors such as income, height, mid-upper arm circumference, alcohol use, and smoking status could not be obtained.

## Conclusion

Residence, occupation, danger signs, and maternal weight during pregnancy were significant determinants of low birth weight. Socioeconomic development, early detection and management of complications, and encouraging nutrition and weight during pregnancy are crucial to minimizing the risk of delivering low birth weight babies. The health care providers would be better to emphasize antenatal counseling and health promotion regarding nutrition and danger signs with the appropriate actions to be taken.

## Data Availability

Data supporting these findings are contained within the manuscript and shared upon request to the corresponding author.

## Abbreviations

ANC: Antenatal care
AOR: Adjusted odds ratio
CI: Confidence interval
COR: Crude odds ratio
EDHS: Ethiopian Demographic and Health Survey
LBW: Low birth weight
NBW: Normal birth weight
SPSS: Statistical Package for Social Science
WHO: World Health Organization
